# Impaired sense of smell and altered olfactory system in RAG-1^−∕−^ immunodeficient mice

**DOI:** 10.3389/fnins.2015.00318

**Published:** 2015-09-09

**Authors:** Lorenza Rattazzi, Anna Cariboni, Ridhika Poojara, Yehuda Shoenfeld, Fulvio D'Acquisto

**Affiliations:** ^1^William Harvey Research Institute, Barts and The London School of Medicine and DentistryQueen Mary University of London, UK; ^2^Department of Pharmacological and Biomolecular Sciences, University of MilanMilan, Italy; ^3^Department of Cell Biology, Institute of Ophthalmology, University College LondonLondon, UK; ^4^Zabludowicz Center for Autoimmune Diseases, Sheba Medical Centre, Sackler Faculty of Medicine, Tel Aviv UniversityTel Aviv, Israel

**Keywords:** immunosuppression, immunodeficiency, anxiety, olfactory dysfunction, main and accessory olfactory system (MOS and AOS), main olfactory epithelium (MOE)

## Abstract

Immune deficiencies are often associated with a number of physical manifestations including loss of sense of smell and an increased level of anxiety. We have previously shown that T and B cell-deficient recombinase activating gene (*RAG-1*)^−∕−^ knockout mice have an increased level of anxiety-like behavior and altered gene expression involved in olfaction. In this study, we expanded these findings by testing the structure and functional development of the olfactory system in *RAG-1*^−∕−^ mice. Our results show that these mice have a reduced engagement in different types of odors and this phenotype is associated with disorganized architecture of glomerular tissue and atrophy of the main olfactory epithelium. Most intriguingly this defect manifests specifically in adult age and is not due to impairment in the patterning of the olfactory neuron staining at the embryo stage. Together these findings provide a formerly unreported biological evidence for an altered function of the olfactory system in *RAG-1*^−∕−^ mice.

## Introduction

Many vertebrates, including most mammals and reptiles utilize the sense of smell for their survival since it is essential for finding nutritious food, a suitable mate and to escape predators (Mombaerts, 2004). Odor perception is a finely regulated process that occurs through a substantial and yet finite number of odorant receptors. Indeed, mammals have 347 genes coding for functional odor receptors among a total of 1000 genes and each olfactory receptor is activated by a specific odorant (Hoover, 2010; Glatz and Bailey-Hill, 2011).

Similar to the olfactory system, the immune system helps us to relate to the external environment and to differentiate harmful or innocuous agents. Recent clinical evidence have suggested that these two systems share more than just a similarity and potentially communicate with each other. Indeed, clinical studies on patients suffering from a wide range of immune disorders such as autoimmune pathologies like multiple sclerosis (Lutterotti et al., 2011; Erb et al., 2012; Garcia-Gonzalez et al., 2013), systemic lupus erythematous (Shoenfeld, 2007; Shoenfeld et al., 2009; Cavaco et al., 2012; Perricone et al., 2013) and Sjögren's syndrome (Midilli et al., 2013) or immunodeficiencies like HIV/AIDS (Graham et al., 1995; Mueller et al., 2002; Vance, 2004, 2007; Vance and Burrage, 2006) have often reported a reduction in threshold of discrimination of different odors (TDI) or olfactory dysfunction typically at the onset of disease. Hence either the absence of adaptive immune cells (as in the case of immunodeficiencies) or the uncontrolled activation of these cells (as in autoimmune diseases) can cause a loss or reduced ability to smell i.e., anosmia or hyposmia.

Over the past few years, we have been investigating the crosstalk between emotions and immune system. Our recent characterization of the emotional behavior of immunodeficient recombination activation gene (*RAG*)-1 knockout mice has revealed an increased level of anxiety-like behavior in these animals. Most interestingly, these changes in behavior were accompanied with changes in the gene expression profile of the brain including a reduced expression of genes involved in olfactory transduction. This was a rather interesting finding considering that the same immunological conditions that cause anosmia have also been associated with increased incidence of mental disorders and stress. Most intriguingly, studies using both pharmacological and surgical impairment of the olfactory system have also described a significant increase in anxiety-like behavior thus suggesting the possibility that smell, anxiety and immune response might share a common molecular pathway.

To test this hypothesis, in this study we investigated if the increased anxiety—like behavior of *RAG-1*^−∕−^ mice was also associated with an impaired sense of smell. In addition to this, because *RAG-1* is expressed in olfactory sensory neurons (OSN) and epithelium (OE) we also investigated if the absence of this gene would affect the development of the olfactory system. Our results provide the first experimental evidence for a specific role of *RAG-1* in the conservation of the olfactory system in adult mice rather than its development at the embryo stage. Most importantly, our studies provide further evidence for a possible role of *RAG-1* as molecular link between emotions, immunity and sense of smell.

## Materials and methods

### Mice

*RAG-1*^−∕−^ mice on C57/BL6 background were kindly provided by Prof. Hans Stauss, (University College London, UK) while control C57BL/6 mice were purchased from Charles River. Both strains were bred in our animal facility. We used 7 week-old male mice for all the behavioral tests and timed-embryos were obtained by mating mice in the evening. The presence of a vaginal plug in the morning indicates successful mating and the resulting embryo would be considered 0.5 day old (E0.5). Mice were housed in groups of maximum 6 animals per cage under specific-pathogen-free conditions and with free access to food and water. All the behavioral experiments were performed during the light phase of the light-dark cycle and no more than 2 tests per day were performed. All tests were conducted in a blinded fashion and according to the UK Animals (Scientific Procedures) Act, 1986. The local biological service unit at Queen Mary University of London approved all experimental protocols.

### Buried food test

First described in the early 1970s (Alberts and Galef, 1971), the buried food test has been adapted under various names and a range of palatable food have been used (e.g., cookies, cereals and food pellets). The purpose of this experimental test is to measure an animal's ability to smell volatile odors and its natural tendency to use olfactory cues for foraging. The main parameters measured in this test are the latency to find the hidden food and the time spent eating it (Yang and Crawley, 2009). The testing protocol of 3 days consists of an odor familiarization exercise on day 1, food deprivation on day 2 and testing on day 3. On day 1, 7-week male C57BL/6 and *RAG-1*^−∕−^ mice were placed in a clean mouse plastic cage (25 × 42 × 12 cm) containing 3 cm of fresh cage bedding. Three Teddy Grahams cookies (Nabisco Inc.; 1 cookie for every 2 mice) where placed in each cage and left overnight. Cages were inspected on day 2 to verify that the cookies were consumed to make sure that the bait is a highly palatable food. On day 2 at approximately 4 pm (1 day before the test), food pellets were removed from the cages and testing mice fasted overnight. The test was performed on day 3 at approximately 11 am after 1-h acclimatization in the testing room. Mice were then individually introduced into a clean cage containing 3 cm deep of clean bedding and allowed to acclimate to the cage for 5 min to reduce the interference of novel environment exploration during the test. A cookie was buried beneath 1 cm of bedding in a random corner of the cage and the mouse introduced into the cage. The site of animal placement and the site at which the cookie was buried remained constant. Time necessary for the animal to retrieve the cookie with its front paws was measured in seconds (latency) up to a maximum parameter of 15 min (900 s was the maximum score; Yang and Crawley, 2009).

### Olfactory habituation/cross-habituation test

The capability of mice to detect and differentiate various odors (social and non-social odors) was examined with the olfactory habituation/cross-habituation test (Luo et al., 2002; Yang and Crawley, 2009). The main aim of this test is to measure an animal's tendency to investigate novel smells and presenting the mice with a sequence of different odors assesses this. A common sequence is (1) water; (2) two non-social odors; and (3) two social odors. Habituation is defined by a decrease in time spent sniffing the same odor. Cross-habituation is represented by a reinstatement of olfactory investigation when a novel odor is presented (Woodley and Baum, 2003; Wrenn et al., 2003; Wersinger et al., 2007). Prior testing, the mice were allowed to acclimate for 30 min to a clean food- and water-deprived testing cage with a dry cotton-tipped applicator inserted through the water bottle hole. This is a necessary practice because it can reduce the interference of novel environment exploration during the olfactory test. Non-social odors were prepared in the morning of the same day of the test, they included: (1) distilled water; (2) solution with almond extract; (3) solution with banana extract (McCormick Inc. brand). Almond and banana are standard non-social odors because they are distinctly different and mildly attractive natural food odors, but unrelated to the food with which laboratory rodents are familiar (Huckins et al., 2013). The solutions were prepared by adding 10 μl of almond or banana extract to 990 μl of distilled water (1:1000 dilution). For the social odors, a cotton-tipped applicator was swiped in a zigzag fashion 5 times on the bottom of a cage. We used 5 day-old dirty cages of female mice for unfamiliar social cage 1 and male mice of the same age for unfamiliar social cage 2. Stimuli were presented in the following order: water × 3, almond × 3, banana × 3, social odor × 3. A trial period of 2 min was given for each stimulus presented, and thus the time spent sniffing the tip for each stimulus was recorded in seconds using a silent stopwatch.

### Immunohistochemistry

Immunofluorescence was applied to formaldehyde fixed cryosections as previously described (Cariboni et al., 2011). Briefly, coronal sections were blocked with serum free protein block (DAKO) and immunostained with goat anti–OMP (1:500; DAKO), rabbit anti-Tuj1 (1:500; Covance), followed by cy3- and 488-conjugated donkey antigoat/rabbit Fab fragment secondary antibodies (Jackson ImmunoResearch). Nuclei were counterstained with DAPI (Sigma). For immunoperoxidase staining, formaldehyde-fixed sections were processed as described previously. Briefly, coronal adjacent sections of formaldehyde-fixed embryo heads of 25 m were incubated with hydrogen peroxide to quench endogenous peroxidase activity and then blocked and incubated with rabbit anti-peripherin (1:1000, Chemicon) or rabbit anti-GnRH (1:1000, ImmunoStar) primary antibodies and followed by biotinylated goat anti-rabbit antibody (Vector Laboratories). Immunoreactivity was visualized with the ABC kit (Vector Laboratories) and 3,3-diaminobenzidine (Sigma). The analysis was performed on at least 3 samples for each genotype. We measured the pixel intensity of OMP staining in 20 μm coronal sections through the olfactory glomeruli of 3 mice for each genotype. Haematoxylin and eosin staining (H&E) was applied to paraffin sections of whole heads as previously described for other tissues (Maione et al., 2009, 2010; Paschalidis et al., 2009). Briefly, whole heads were obtained from 7-week old wild-type C57BL/6 and *RAG-1*^−∕−^ mice. Prior fixation with 4% paraformaldehyde (pH 7.4) and decalcification in 10% EDTA (pH 7.2–7.4), the heads were embedded in paraffin wax. Sagittal sections were deparaffinized and stained with haematoxylin and eosin. Digital images were taken using the Image Pro image analysis software package.

### Data analysis

All the statistical analysis was performed using GraphPad Prism software. The buried food test was analyzed using the nonparametric Mann–Whitney *U*-test. Statistical significance was set at *p* ≤ 0.05 and all data are presented as mean ± SEM as previously described (Dawson et al., 2005; Fleming et al., 2008). For the habituation/dishabituation test, One-Way repeated measured ANOVA within each group was used to compare the time that subjects spent investigating the stimulus upon the different exposure. All data are presented as mean ± SEM.

The total number of GnRH neurons/head was quantified as previously described (Cariboni et al., 2011). To compare the abundance of OMP^+^-neurons we measured the pixel intensity of OMP staining in 20-μm coronal sections through the OB of 3 mice for each genotype, by using ImageJ software (NIH). To compare the area of glomeruli, we measured the area of each glomerulus in 20-μm coronal sections through the OB of adult mice for each genotype, at the same anatomical level, by using ImageJ software. To compare the OE thickness, we measured the thickness of OE in 10-μm sagittal sections through the nasal region for each genotype, at the same anatomical level, by using ImageJ software. To determine statistical significance, we used the unpaired *t*-test. A *P*-value of less than 0.05 was considered statistically significant. For all experiments, data are expressed as the mean ± SEM.

## Results

### *RAG-1*^−∕−^ immunodeficient mice have an altered sense of smell

**Figure 1:**
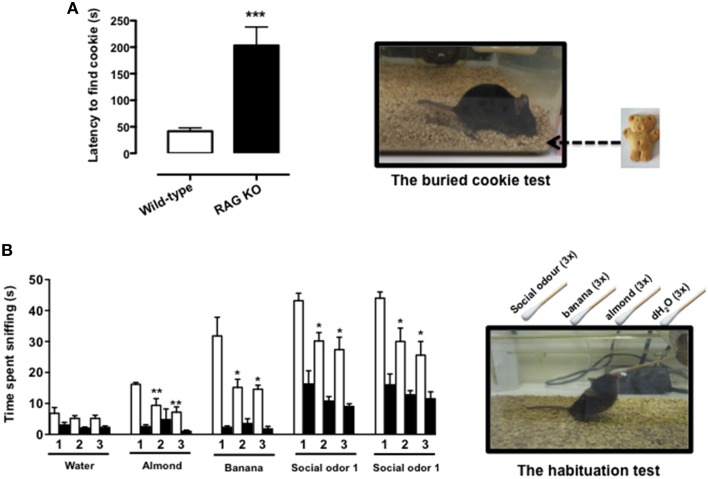
*****RAG-1***^−∕−^ mice show an impaired sense of smell**. Adult 7 week-old *RAG-1*^−∕−^ and control C57/BL6 mice were tested with the buried cookie test (top panels) or the habituation/dishabituation test (bottom panels) as described in Materials and methods. The bar graph in **(A)** represents the time expressed in seconds required to find the buried cookie. Values are mean ± SEM obtained from a single experiment with *n* = 5 mice and representative on *n* = 4 experiments with similar results. ^***^p < 0.005 vs. C57BL/6 control mice. The graph in **(B)** shows the time expressed in seconds spent sniffing the stimuli (water, almond, banana, and social odor). The numbers on the x-axes (1, 2, and 3) indicate the order of the repetitive exposure i.e., 1st, 2nd, and 3rd. Values are mean ± SEM obtained from a single experiment with *n* = 5 mice and representative on *n* = 3 experiments with similar results. ^*^p < 0.05; ^**^p < 0.01 vs. the 1st exposure. The left top and bottom pictures show a schematic representation of the buried cookie test (top) and the habituation/cross-habituation test (bottom) described in details Materials and Methods Section.

The buried food test is a reliable protocol that relies on the natural tendency of the mouse to use olfactory cues for foraging. The main parameter is the latency to uncover a small piece of palatable food such as a cookie, hidden beneath a layer of sawdust, within an established length of time. We first tested the palatability of the bait leaving the cookie with mice overnight (see Materials and Methods Section) and observed no difference between *RAG-1*^−∕−^ and control C57/BL6, i.e., both strains consumed the whole cookie. However, when we performed the test of the buried cookie, we observed a significant five-fold increase in the latency to find the bait (203 s ± 77.7 vs. 42 s ± 18.9; *p* < 0.001) in the *RAG-1*^−∕−^ compared to control C57/BL6 mice (Figure 1A).

To further assess the olfactory function of *RAG-1*^−∕−^ mice, we used the habituation/cross-habituation test, which relies on the animal's tendency to explore novel smells and is also used to evaluate its ability to distinguish between different odors (Yang and Crawley, 2009). When presented with different stimuli (water, almond, banana, and social odor), control C57BL/6 mice (wild-type) showed the expected increase in time sniffing (compare number 1 bar in the water group with number 1 bar in the other groups) every time a new odor was introduced (cross-habituation). They also showed habituation to the same stimuli since the time spent sniffing the same stimuli was significantly reduced upon the second and the third exposure (compare white bar 1 with bars 2 and 3 in each group) (Figure 1B). In contrast, *RAG-1*^−∕−^ mice did not follow this pattern and showed an overall reduction in the time of sniffing. More specifically, the investigation rate was so low that both habituation and cross–habituation were difficult to be assessed. Interestingly, however, *RAG-1*^−∕−^ mice showed an increase in the time spent exploring the social stimulus compared to unfamiliar ones (almond and banana) and showed a trend toward a normal pattern of habituation (although the differences between the 3 exposure were not significant) suggesting a preserved function of the vomeronasal organ and an impaired activity of the main olfactory system (see Discussion).

### Histological assessment of the olfactory and vomeronasal systems in *RAG-1*^−∕−^ embryos

**Figure 2:**
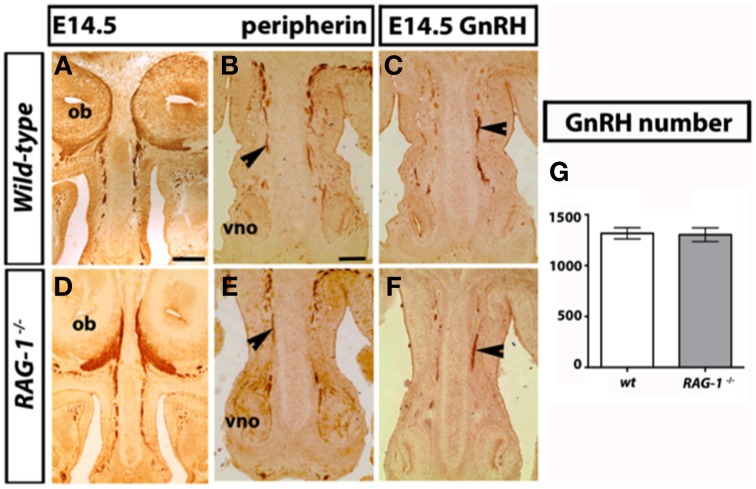
**Normal GnRH neuron development in embryonic ***RAG-1***^−∕−^ mice**. Coronal sections of embryonic day E14.5 mouse heads *RAG-1*^−∕−^ and control C57/BL6 mice were stained for peripherin **(A,B,D,E)** or GnRH **(C,F)** as described in Material and Methods. Black arrowheads indicate examples of extending olfactory/vomeronasal axons **(B,E)** and of migrating GnRH neurons in the nasal area **(C,F)**. Quantitation of total GnRH neuron number is displayed in **(G)**. Pictures are representative of *n* = 4–6 mice of each genotypes. Scale bars: 150 μm **(A,D)**; 100 μm **(B,C,E,F)**.

The development of the olfactory system is strictly linked to the development of the gonadotropin-releasing-hormone neurons, which regulate reproductive function (Wray, 2010; Forni and Wray, 2014). These neuroendocrine cells originate in the nasal placode, the embryonic structure that gives rise to the OE and VNO, and migrate toward the brain apposed to olfactory (OLF) and vomeronasal (VN) axons. To investigate the mechanisms behind the olfactory deficits observed in the *RAG-1*^−∕−^ mice, we first analyzed the development of the main and accessory olfactory systems at day 14.5 (E14.5) by staining coronal head sections with an anti-peripherin and anti-GnRH antibodies as previously described (Cariboni et al., 2011). As shown in Figure 2, we did not observe any defects in either the fasciculation or the targeting of the olfactory nerves toward the olfactory bulbs (OB) between wild-type and *RAG-1*^−∕−^ embryos (Figures 2A,D, respectively). In addition, the vomeronasal nerves, responsible for pheromone detection in adulthood, were normal and comparable between the two genotypes (Figures 2B,E) as it was the migration and the number of the gonadotropin-releasing hormone neurons responsible for reproduction (Figures 2C,F,G; total number of GnRH neurons: wild-types 1317 ± 27.55 vs. *RAG-1*^−∕−^ 1303 ± 33.37, *p* = 0.76). Consistent with this, the size of the gonads in 7 week-old mice showed no gross difference between wild-type and *RAG-1*^−∕−^ (Supplementary Figure 1, respectively).

### Histological analysis of the olfactory bulbs of newborn *RAG-1*^−∕−^ mice

**Figure 3:**
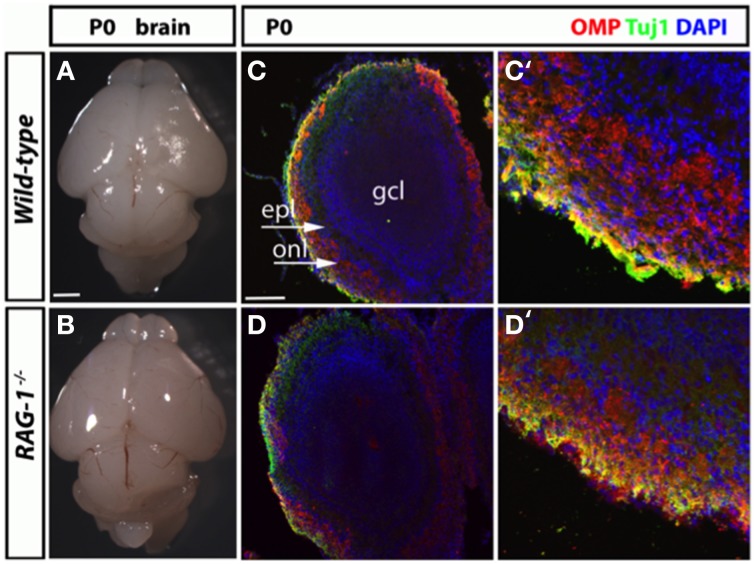
**Histological analysis of the olfactory system in newborn ***RAG-1***^−∕−^ mice. (A,B)** Brains from male postnatal age (P) 0 newborn *RAG-1*^−∕−^ and control C57/BL6 mice were photographed side-by-side to demonstrate no differences in the size and gross morphology of the brain and of the olfactory bulbs. **(C,D)** Coronal sections of the olfactory bulb from the same mice immunostained with antibodies against OMP and Tuj1 revealed no differences in the intensity and morphology of the projecting ORNs. DAPI was used to counterstain the nuclei. **(C**′**,D**′**)** Show higher magnifications of the same tissue sections. The organization of the olfactory bulb displayed in **(C,D)** are labeled as following: gcl, granule cell layer; epl, external plexiform epithelial; onl, olfactory nerve layer. Pictures are representative of *n* = 3 mice of each genotypes. Scale bars: 1 mm **(A,B)**; 100 μm **(C,D)**.

We next conducted a histological assessment of the olfactory system at birth (day 21) just before the pups are exposed to external and social stimuli. Analysis of the size and gross morphology of the olfactory bulbs in newborn *RAG-1*^−∕−^ mice showed no differences compared to control wild-type mice (Figures 3A,B, respectively). Immunostaining of the same tissues for the olfactory marker protein OMP and the pan-neuronal precursor marker Tuj1 confirmed these results and showed no difference in the localization or level of expression of these two markers (Figures 3C,D).

### Impaired olfactory system in adult *RAG-1*^−∕−^ mice

**Figure 4:**
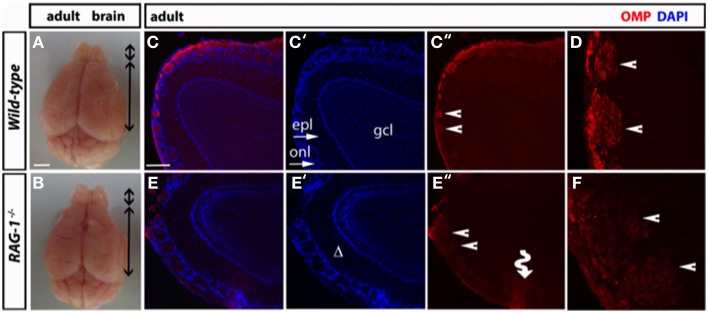
**Impaired tissue structure of the olfactory system of adult ***RAG-1***^−∕−^ mice. (A,B)** Brains from male postnatal age (P) 21 *RAG-1*^−∕−^ and control C57/BL6 mice were photographed side-by-side to demonstrate no differences in the size and gross morphology of the brain and of the olfactory bulbs (OB). **(C,D)** Coronal sections of the olfactory bulb from the same mice immunostained with an anti-OMP antibody showed a reduced OMP signal in the mutant mice compared to controls. DAPI was used to stain the nuclei. The funny arrow **(E**″**)** highlights the disorganized structure of the glomerulus in the mutant OB. **(D,F)** are higher magnification of the areas pointed by the arrows in **(C**″**,E**″**)**. Pictures are representative of *n* = 3 mice of each genotypes. Scale bars: 1 mm **(A,B)**; 100 μm **(C,E)**.

Morphological analysis of the olfactory bulb of fully developed (7 week-old) *RAG-1*^−∕−^ adult mice did not present any differences in size compared to control age-matched wild-type (Figures 4A,B, respectively). However, immunofluorescene staining of the olfactory bulb for OMP showed disorganized glomeruli (which are the initial sites for synaptic processing of odor information coming from the nose, Zou et al., 2009; Sakano, 2010) (Figures 4C,E) as well reduced expression of this marker (Figures 4D,F). Quantitative analysis of OMP staining over different sections confirmed these results and showed a significant (*p* < 0.01; *n* = 3) reduction of about 50% of the pixel intensity in *RAG-1*^−∕−^ mice (15.9 ± 1.7 mean pixel intensity/area) compared to wild-type control (28.8 ± 2.3 mean pixel intensity/area). No differences were observed in the size of each glomerulus, expressed as mean of the area (wild type: 0.0224 ± 0.0017 vs. *RAG-1*^−∕−^ 0.0255 ± 0.0022, *p* = 0.72; Area expressed as square mm).

**Figure 5:**
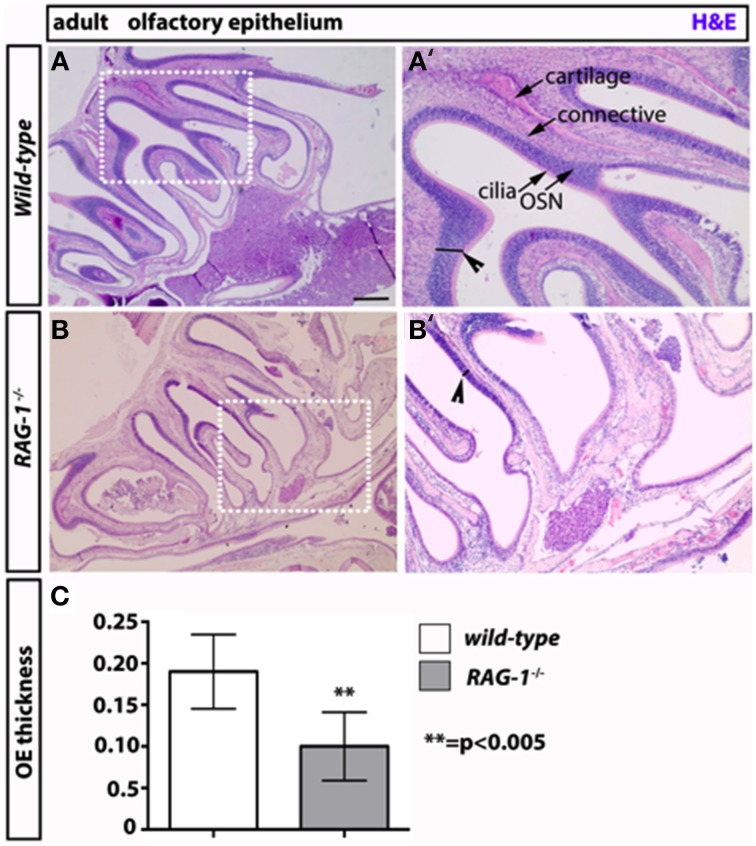
**Decreased thickness and cellularity of the main olfactory epithelium of ***RAG-1***^−∕−^ mice**. The pictures in **(A,B)** show the hematoxilin and eosin staining of the MOE of 7 week-old *RAG-1*^−∕−^ and control C57/BL6 mice. The pictures in **(A′,B′)** are higher magnification of the boxed areas in **(A,B)** and highlight the differences in thickness (black segments) between the two tissues (arrowheads). The different cellular layers of MOE are indicated with arrows and correspond to: cilia, chemosensory cilia; OSN, olfactory sensory neurons; connective, connective tissue and cartilage. Quantitation if OE thickness is displayed in **(C)**. Pictures are representative of *n* = 3 mice of each genotypes. Scale bars: 150 μm **(A,B)**.

On the opposite site of the glomeruli, olfactory neurons innervate the olfactory epithelium (Leinwand and Chalasani, 2011; Murthy, 2011; Takeuchi and Sakano, 2014). These tissues present in the turbinates of the nose act as “platform” for the olfactory neurons and undergo continuous regeneration. Given that olfactory bulbectomy has been shown to severely affect olfactory epithelium regeneration (Suzuki et al., 1998; Makino et al., 2009), we reasoned that the absence of fully functional olfactory neurons would impact the status of OE in *RAG-1*^−∕−^. Consistent with our expectation, staining of sagittal paraffin sections with haematoxylin and eosin showed reduced cellularity and thickness of the MOE in *RAG-1*^−∕−^ tissues compared to wild-type control (Figures 5A,B, respectively; Figure 5C, OE thickness: wild type 0.1900 ± 0.01581 vs. *RAG-1*^−∕−^ 0.1000 ± 0.01558; *p* < 0.005) further supporting the idea that the absence of immune cells may cause histological changes in olfactory neurons and an impairment of olfaction mainly in adult mice.

## Discussion

Immunodeficiencies have long being associated with a number of physical manifestations that are not generally linked to immune functions including anxiety and anosmia. Performing a study on the behavioral profile of T and B cell-deficient *RAG-1*^−∕−^ mice we have observed an increased level of anxiety-like and surprisingly found significant changes in brain gene expression profiles of these mice when compared with their wild-type littermates. Pathways analysis of these genes revealed a number of interesting links to different diseases and unexpectedly a defect in the signaling pathways involved in the olfactory system. This was a rather interesting finding since it suggested that *RAG-1*^−∕−^ mice could represent an ideal experimental system to study the simultaneous occurrence of anxiety and anosmia that has been described in clinical cases of immunodeficiency.

To verify our hypothesis, we first tested the olfactory function of *RAG-1*^−∕−^ mice using classical behavioral models based on the ability of the mouse to recognize the odor of “palatable” baits. Our results from the buried cookie tests showed an increase in the latencies to find the buried food and recognizing the stimulus in *RAG-1*^−∕−^ mice compared to control. These differences in odor recognition were further confirmed with the habituation/cross-habituation test where the *RAG-1*^−∕−^ mice showed an impaired pattern of habituation and cross-habituation to distinct and yet volatile odors.

As common feature of both tests, *RAG-1*^−∕−^ mice showed an overall difficulty in preforming the expected task provided (finding the cookie or being interested in different odor stimulations) and this might be linked to their increased level of anxiety-like behavior that makes them distracted from the task. Consistent with this, other authors have shown an increased locomotor and exploratory activity and degree of anxieties in chemically induced anosmic mice (Kudyakova et al., 2007; Glinka et al., 2012).

Aiming to further understand the cellular mechanism behind the impaired sense of smell of *RAG-1*^−∕−^ mice, we investigated whether there were any defects in the cellular structure of the olfactory organs at three key time points: at the embryo stage, soon after birth and at adult age (6–8 weeks). Our results show that *RAG-1*^−∕−^ had no differences in the development of the olfactory nerves at embryonic day 14.5, which is the stage of development that follows the establishment of the first olfactory sensory link between the olfactory epithelium and the olfactory bulb. Same results were obtained in the expression of key olfactory markers OMP and Tuj1 in the olfactory bulb at day 0 suggesting no involvement of *RAG-1*^−∕−^ in the development of the olfactory system at this stage. Indeed, although we did not perform any behavioral test for olfaction in newborn mice, at observational level we did not see any difference in the ability of the newborn *RAG-1*^−∕−^ to recognize the nipples of the lactating mother. Nor we observed any difference in the weight of these pups that would suggest an impaired ability to feed themselves because of olfactory defects (data not shown).

Histological analysis of the MOE in adult mice provided us with a completely different scenario featured by a significant reduction in thickness and cellularity of the epithelium and a disorganized architecture of the glomerular tissue of the olfactory bulb in *RAG-1*^−∕−^ mice compared to control C57BL/6 mice. These structural differences might explain the increased time needed to track the volatile odor released by the chocolate chip cookie in *RAG-1*^−∕−^ mice. The MOE is largely tasked with smelling inherently “neutral odors” (Munger, 2009; Huckins et al., 2013) and its dysfunction can be readily observed in anosmic mice that are known to typically display a significant reduction in the latency to identify an odor stimulus. Most interestingly, the changes across the MOE might provide an explanation for the increased anxiety behavior of *RAG-1*^−∕−^ mice that we have previously reported (Rattazzi et al., 2013). Congruently, recent studies have suggested that “*functional activation of the MOE but not the VNO causes elevated levels of anxiety*” (Glinka et al., 2012). The reverse might also be true since studies in humans have also suggested that the induction of a state of anxiety provoke a shift in the perception of a neutral odor (that becomes unpleasant) and to an increase in time needed to detect it (Krusemark et al., 2013).

We did not find any defect in the structure of the VNO (data not shown) and this might also explain the unperturbed response of *RAG-1*^−∕−^ mice to social odor. These results are consistent with previous studies by Mcgowan et al. (2011) where the authors described an intact ability of *RAG-1*^−∕−^ mice to recognize social odor. The same study, however, differs from our as the authors have found no changes in the recognition of non-social odors by *RAG-1*^−∕−^ mice. The differences in the results are most likely due to the different experimental settings they used i.e., high volatile odors (lemon and peppermint instead of almond and banana) and much older mice (3–5 months as opposed to 7–8 week old). In addition to this, in this study the authors showed no difference in anxiety-like behavior in the open field test while previous results from our and other research groups showed significant difference in open field, marble burying and light/dark box. We do not know how to explain these discrepancies except with possible differences in the housing conditions and gut microbiota that could account for difference in behavior.

The current study does not establish if the defects in olfaction of the *RAG-1*^−∕−^ are due to the lack of immune system or to an intrinsic role of *RAG-1* gene in the development of the olfactory system. Previous studies have shown that RAG-1 is expressed in the olfactory neurons as well as in other brain regions such as cerebellum and the hippocampal of mice (Chun et al., 1991)—in the olfactory neurons contained in the two placodes located anterior and dorsal to the eyes in zebrafish (Jessen et al., 1999, 2001)—and in a subpopulation of zebrafish olfactory neurons projecting to the lateral olfactory bulb (Feng et al., 2005). Consistent with our findings, the study performed in zebrafish showed that depletion of RAG-1 by morpholino-mediated knockdown or mutation, did not affect axon targeting. If we combine these observations together, it is possible to exclude that RAG-1 plays a key role in OSN development during the embryo stage.

Looking at the adult stage, our results differ from those obtained in zebrafish since in these animals there was no changes of odorant receptor expression or response of OSNs to amino acids. We do not know the reason behind this discrepancy and we are tempted to think that T cells might be responsible for the changes in odor perception. The full validation of this hypothesis would require a full new set of investigations addressing a number of specific questions. Nevertheless, we think that there are considerations that can be taken into account in support of this idea.

First of all, our previous study on the emotional behavior of *RAG-1*^−∕−^ mice showed that *RAG-1*^−∕−^*/OT-II* but not *RAG-1*^−∕−^*/OT-I* could “rescue” the gene expression profile and behavior of the former (Rattazzi et al., 2013). This suggested to us that T cells rather than B cells, and CD4^+^ T cells (present in *RAG-1*^−∕−^*/OT-II*) rather CD8^+^ T cells (present in *RAG-1*^−∕−^*/OT-I* mice), had a significant impact on the observed impaired emotional behavior of *RAG-1*^−∕−^ mice. Looking at the changes in olfactory transduction pathway of these very same mice, a similar patter of regulation could be observed i.e., *RAG-1*^−∕−^*/OT-II* were similar to wild type supporting the idea that CD4^+^ T cells might rescue the olfactory defect of *RAG-1*^−∕−^ mice(Rattazzi et al., 2013).

In terms of the mechanism by which CD4^+^ T cells would control the healthy state of the MOE, we speculate that this might be linked to the impaired development of nasal-associated lymphoid tissue (NALT) (Bienenstock and Mcdermott, 2005; Ruddle and Akirav, 2009). Like all other mucosal-associated lymphoid tissues, these are organized clusters of T and B cells that act as patrolling stations and local reservoir of the immune system in the mucosal districts. Studies on the origin of NALT have shown these structures develop before the other secondary lymphoid organs and start at postneonatal age continuing till weaning (Bienenstock and Mcdermott, 2005; Drayton et al., 2006; Ruddle and Akirav, 2009; Brandtzaeg, 2011). Most interesting, the structural differentiation of the NALT has been proposed to be completed after 6 weeks of age (which is the age of the mice we have used in our tests) and to be influenced by environmental stimuli. Given that the presence of these structures has been shown to be important for the release of factors regulating olfactory epithelium proliferation, differentiation, and maturation (Schwob, 2002), it would be tempting to assume that their absence is one of the main causes of atrophy (reduced thickness and cellularity) of the MOE of the *RAG-1*^−∕−^ mice and, as consequence of that, of the impaired organization of the glomeruli in the olfactory bulb.

In conclusion, the results of this study provide first evidence for an impaired olfactory function in adult *RAG-1*^−∕−^ mice. Future studies using this animal model might help to identify new therapeutic targets or experimental approaches to investigate possible link between immunodeficiency, anxiety and anosmia.

## Author contributions

LR and RP performed the behavioral experiments and the collection of tissues. LR and AC performed the histological analyses and helped writing and revising the manuscript. FD and YS designed the study, analyzed the data, and wrote the manuscript.

### Conflict of interest statement

The authors declare that the research was conducted in the absence of any commercial or financial relationships that could be construed as a potential conflict of interest.

## Supplementary material

Supplementary Figure 1 **Normal morphology of the gonads of adult *RAG-1^−/−^* mice**. Testes pairs from adult littermate males of the indicated genotypes were photographed side-by-side to show no differences in the size and gross morphology of the gonads of 7 week-old *RAG-1^−/−^* and control C57/BL6 mice. Pictures are representative of *n* = 4–6 mice of each genotypes. Scale bar: 500 μm.
