# Supplementary figures and images for: Impaired sense of smell and altered olfactory system in RAG-1−∕− immunodeficient mice
Source: Front Neurosci. 2015 Sep 9;9:318. doi: 10.3389/fnins.2015.00318 (PMC4563081; doi:10.3389/fnins.2015.00318)

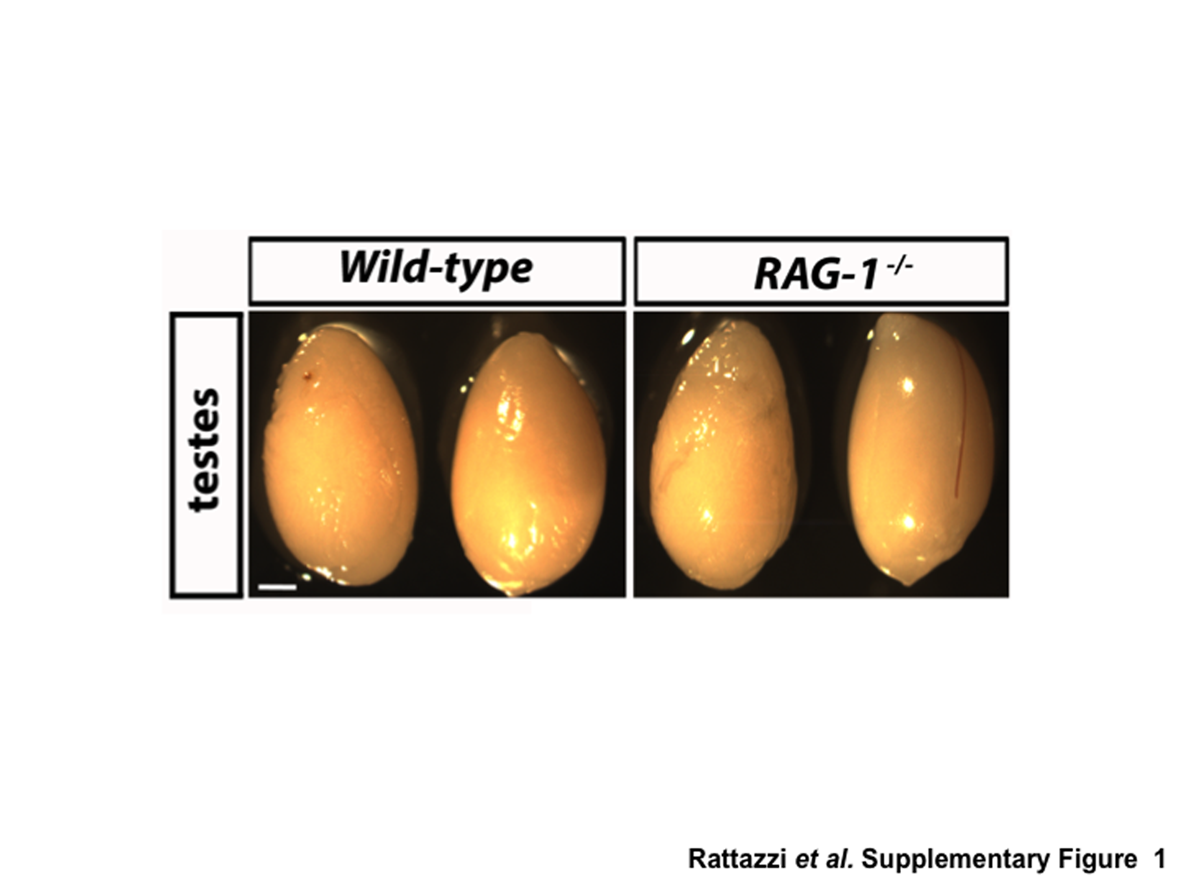

Supplement: Supplementary Figure 1 — Normal morphology of the gonads of adult RAG-1−/− mice. Testes pairs from adult littermate males of the indicated genotypes were photographed side-by-side to show no differences in the size and gross morphology of the gonads of 7 week-old RAG-1−/− and control C57/BL6 mice. Pictures are representative of n = 4–6 mice of each genotypes. Scale bar: 500 μm. [file Image1.TIFF]
